# Exercise Enjoyment and Exercise Addiction Risk Among Turkish Adults: Associations and Subgroup Differences in a Cross-Sectional Survey

**DOI:** 10.3390/healthcare14060703

**Published:** 2026-03-10

**Authors:** Bekir Erhan Orhan, Hussain Yasin, Aydın Karaçam, Walaa Jumah AlKasasbeh, Mehdi Ben Brahim

**Affiliations:** 1 Faculty of Sports Sciences, Istanbul Aydın University, Istanbul 34295, Türkiye; 2 Health and Physical Education (HPE) Department, Sport Sciences and Diagnostics (GSD) Research Group Department, Prince Sultan University, Riyadh 11586, Saudi Arabia; hyasin@psu.edu.sa; 3 Faculty of Sports Sciences, Bandırma Onyedi Eylül University, Balıkesir 10200, Türkiye; akaracam@bandirma.edu.tr; 4 Department of Physical Education, School of Sports Sciences, The University of Jordan, Amman 11942, Jordan; walaakasasbeh1991@yahoo.com

**Keywords:** exercise enjoyment, exercise addiction risk, compulsive exercise, exercise motivation, physical activity

## Abstract

**Background:** Exercise enjoyment supports adherence, whereas elevated exercise addiction risk reflects potentially maladaptive persistence marked by rigidity and internal pressure. This study examined the association between enjoyment and exercise addiction risk in Turkish adults and explored variation across sociodemographic, lifestyle, and exercise-related characteristics. **Methods:** A total of 420 adults (45.0% women, 55.0% men; mean age = 25.68 years) completed an online survey including the Exercise Enjoyment Scale (EES) and the Exercise Addiction Inventory (EAI). **Results:** Enjoyment was weakly and inversely associated with exercise addiction risk (*r* = −0.18, *p* = 0.0002; 95% CI: −0.27 to −0.09). Women reported higher enjoyment and higher EAI scores than men. The proportion screening positive for elevated risk (EAI ≥ 24) was 13.8% (*n* = 58; 95% CI: 10.8–17.4%); subgroup comparisons were interpreted as exploratory (no multiplicity correction). **Conclusions:** Enjoyment tended to vary with participation patterns, whereas addiction risk tended to vary with training structure and motives; longitudinal studies are needed to clarify temporal ordering.

## 1. Introduction

Regular physical activity is a central pillar of adult health, yet the most persistent challenge in real-world settings is sustaining participation over time [1,2]. Participation commonly declines when individuals encounter time pressure, stress, fatigue, or low perceived competence, especially when exercise experiences feel unrewarding [3,4]. These patterns indicate that sustained activity is shaped not only by access and intentions but also by the psychological experience of exercise; how it feels, what it delivers in the moment, and how it fits into everyday life [5,6,7].

Within this experiential framework, exercise enjoyment is particularly important because it captures the immediate reward value of exercise [8,9]. Enjoyment reflects the extent to which exercise is experienced as pleasurable, satisfying, and engaging, and it is consistently linked to persistence because rewarding experiences encourage repetition and support routine formation [2,10]. From a self-determination theory perspective, enjoyment is more likely to be sustained when exercise contexts support basic psychological needs (autonomy, competence, and relatedness), thereby strengthening self-endorsed regulation and long-term adherence [11,12,13]. Accordingly, program features such as autonomy-supportive instruction, preference-matched activity selection, appropriate intensity progression, competence-building feedback, and supportive social climates can improve affective experiences of exercise and support maintenance [11,12].

At the same time, sustained exercise is not uniformly adaptive. Exercise addiction risk refers to a pattern of rigid and compulsive exercise involvement in which exercise becomes disproportionately central, difficult to regulate, and maintained despite potential costs (e.g., reduced flexibility, distress when unable to exercise, interference with recovery or life roles) [14,15]. In community research, this construct is typically assessed via screening measures that indicate elevated risk rather than clinical disorder status; nevertheless, identifying risk tendencies is important because they can undermine well-being even when total activity levels appear “healthy” by conventional standards [15,16]. Theory and emerging evidence suggest that maladaptive persistence is more closely linked to controlled forms of regulation and need frustration than to enjoyment alone, highlighting that high exercise volume can coexist with qualitatively different motivational processes [13,17].

Considering enjoyment and addiction risk together is therefore critical because both relate to persistence, but they may reflect different regulatory pathways. Enjoyment-driven participation is typically characterized by flexible self-regulation: individuals continue because exercise is rewarding and meaningful and can adjust training without marked distress [6,18]. In contrast, addiction-like persistence may be maintained by internal pressure, compensatory motives (e.g., to control weight or cope with stress), or identity-contingent standards, allowing exercise to continue even when positive affect is reduced [14,19]. Harmonious forms of engagement are generally compatible with flexibility and well-being, whereas obsessive forms are more likely to involve rigid persistence and conflict with other life domains; therefore, enjoyment and addiction risk may be inversely associated, but only modestly so because enjoyment and rule-governed involvement can co-occur [14,20,21,22]. Both constructs may also vary across sociodemographic, lifestyle, and exercise-related profiles (e.g., gender, age, sleep patterns, frequency, and motives), which can shape exposure to appearance/performance pressures, routine stability, and the meaning of structured training in daily life [11,23,24,25,26,27].

Studying these dynamics in Turkish adults is particularly relevant given the public health importance of adult activity patterns and weight-related outcomes and the sociocultural contexts that may shape exercise motives and body-related standards. Accordingly, this study examined the association between exercise enjoyment and exercise addiction risk in Turkish adults and explored whether both constructs differed across sociodemographic, lifestyle, and exercise-related characteristics. The study hypothesized a small inverse association between enjoyment and addiction risk and expected both constructs to vary across exercise participation profiles and motives.

## 2. Materials and Methods

### 2.1. Study Design

This study used a descriptive, cross-sectional correlational survey design to examine the association between exercise enjoyment and exercise addiction risk and to explore variation across selected demographic, lifestyle, and exercise-related characteristics [28]. The study was planned and reported in line with STROBE guidance for cross-sectional studies [29]. Because all variables were measured at a single time point and were not manipulated, analyses estimate associations and do not establish causal direction or causal effects [30].

### 2.2. Participants

Participants were recruited using convenience sampling, an accessibility-based approach that enables rapid recruitment [31]. In total, 420 adults aged ≥ 18 years completed the online survey and were included in the analyses. Eligibility criteria were: age ≥ 18 years, ability to read Turkish, and provision of electronic informed consent. Exclusion criteria were: non-completion of the questionnaire (i.e., missing key study variables) and suspected duplicate submissions. The survey link was disseminated via email lists and social media platforms (e.g., Instagram and WhatsApp groups) and through university/community clubs and groups. The form settings were configured to minimize duplicate submissions (e.g., one-response-per-account setting and screening for duplicate response patterns). Items were set as required; therefore, item-level missingness was minimal. As an online, non-probabilistic convenience sample, participation was self-selected and may not represent all Turkish adults.

### 2.3. Ethical Approval and Informed Consent

This study received approval from the Istanbul Aydın University Social and Human Sciences Ethics Committee (Meeting No.: 2025/12). All procedures were conducted in accordance with the Declaration of Helsinki and relevant institutional guidelines. Participation was voluntary; all participants provided electronic informed consent prior to completing the online survey.

### 2.4. Measures

#### 2.4.1. Exercise Enjoyment Scale (EES)

To assess exercise enjoyment, the Exercise Enjoyment Scale (EES)-an 8-item short form originally developed in a 7-point bipolar format [32] and adapted into Turkish [33]-was used. The instrument consists of eight items rated on a seven-point bipolar Likert-type scale and has a single-factor structure designed to assess enjoyment during exercise. Items 1, 4, 6, and 8 are reverse-coded; higher total scores indicate greater enjoyment of physical activity [33]. In the Turkish adaptation, model fit was mixed: incremental fit indices were high (CFI/IFI ≈ 0.94–0.96) and SRMR was low (≈0.045–0.048), whereas RMSEA was elevated (0.085–0.097) [33]. Internal consistency reliability (Cronbach’s alpha) was reported as 0.87 for the adolescent form and 0.82 for the adult form [33]. In the present study, internal consistency was Cronbach’s alpha = 0.87 and McDonald’s omega = 0.87 for the EES total score. Consistent with the established single-factor structure, analyses used the EES total score; additional factor-analytic revalidation was not performed in this sample.

#### 2.4.2. Exercise Addiction Inventory (EAI)

To assess exercise addiction risk, the Exercise Addiction Inventory (EAI), originally developed by Terry, Szabo, and Griffiths [34] and adapted into Turkish by Aydın et al. [35], was used. The scale consists of six items with a single-factor structure and is designed to screen for elevated risk of exercise addiction (i.e., it indicates risk tendencies rather than a clinical diagnosis). Items were rated on a 5-point Likert-type frequency scale ranging from “Never” (1) to “Always” (5), with higher total scores indicating greater addiction risk. Total scores range from 6 to 30, and scores ≥ 24 are commonly used to indicate elevated risk [34,35]. In the present study, the EAI demonstrated good internal consistency (Cronbach’s α = 0.81; McDonald’s ω = 0.81). Consistent with the established single-factor structure, analyses used the EAI total score; additional factor-analytic revalidation was not performed in this sample.

### 2.5. Statistical Analysis

Data were screened for inaccurate entries, outliers, and distributional assumptions. No incorrectly entered data were detected. All analyses were conducted using SPSS 27 (IBM Corp., Armonk, NY, USA). Descriptive statistics were computed for all variables. Distributional assumptions were evaluated using skewness and kurtosis coefficients and visual inspection of histograms and Q–Q plots. Because distributions were judged acceptable for parametric analyses, independent-samples *t*-tests (two-group comparisons) and one-way ANOVA with Tukey post hoc tests (≥3 groups) were used. Pearson product–moment correlations were used for continuous associations. Given the number of subgroup comparisons, tests were treated as exploratory and interpreted cautiously; no formal adjustment for multiple comparisons was applied, and emphasis was placed on effect sizes and consistency of patterns. For transparency, 95% confidence intervals (CIs) are reported for the primary EES–EAI correlation and for the prevalence estimate based on the EAI cut-off. Effect sizes were reported (Cohen’s *d* for *t*-tests, eta squared η^2^ for ANOVA, and Pearson’s r for correlations) and interpreted using conventional benchmarks [36]. McDonald’s omega (ω) coefficients were computed using an auxiliary reliability procedure compatible with SPSS and are reported alongside Cronbach’s alpha to complement a tau-equivalence-dependent index. An a priori power analysis was conducted using G*Power 3.1.9.2 (Franz Faul, Universität Kiel, Germany) for a two-tailed correlation (α = 0.05, power = 0.95, |*r*| = 0.18), yielding a required sample size of *N* = 396; the final sample (*N* = 420) exceeded this threshold [37,38]. To address potential confounding, a univariate general linear model (GLM; ANCOVA) modeled exercise enjoyment (EES total score) as the dependent variable and included exercise addiction (EAI total score) and age as covariates, with gender, BMI category, and exercise frequency entered as fixed factors. Model assumptions were evaluated using residual diagnostics. Enjoyment (EES total score) was modeled as the dependent variable and exercise addiction risk (EAI total score) as a key predictor, alongside covariates, to provide a covariate-adjusted estimate of the association between enjoyment and exercise addiction risk; the model is intended for adjusted association (not causal direction).

### 2.6. Demographics and Exercise Characteristics

Demographic and exercise-related information was collected via an online self-report form. Participants reported age (years), gender (female/male), marital status (married/single), employment status (employed/unemployed), and highest completed education (high school/bachelor’s/postgraduate). Exercise characteristics were recorded using predefined response categories: sleep status (regular/partly regular/irregular), alcohol use (uses/does not use/occasionally), exercise frequency (<1 day/week; 1–2 days/week; 3–4 days/week; 5–7 days/week), exercise history (0–1 year; 1–3 years; 3–5 years; >5 years), and single-session exercise duration (0–30 min; 31–60 min; 61–90 min; >91 min). Participants additionally selected their primary exercise type (cardio; strength training; flexibility-based) and primary exercise motive (weight loss/control; performance enhancement; health and fitness; stress management; socialization). BMI (kg/m^2^) was calculated from self-reported body weight (kg) and height (m), and participants were classified as underweight (<18.5), normal weight (18.5–24.9), overweight (25.0–29.9), or obese (≥30.0). Several contextual variables (e.g., sleep regularity and alcohol use) were measured using brief categorical items; therefore, subgroup comparisons should be interpreted as descriptive and potentially sensitive to misclassification.

## 3. Results

### 3.1. Participant Characteristics and Prevalence of Elevated Exercise Addiction Risk

Participant characteristics are presented in Table 1.

Based on the commonly used EAI cut-off (≥24), 58 participants (13.8%; 95% CI: 10.8–17.4%) screened positive for elevated exercise addiction risk. Subgroup comparisons are presented with effect sizes and should be interpreted as exploratory.

### 3.2. Sociodemographic Differences in Exercise Enjoyment and Exercise Addiction Risk

Women reported higher exercise enjoyment (small effect; d = 0.24) and higher exercise addiction-risk scores (moderate effect; *d* = 0.55) than men (Table 2). Marital status was not associated with exercise enjoyment or exercise addiction-risk scores (Supplementary Table S1). Employment status was not associated with exercise enjoyment or exercise addiction-risk scores (Supplementary Table S2).

Table 3 indicates education level was associated with EES (*F* = 4.23, *p* < 0.05, η^2^ = 0.02) and EAI (*F* = 31.69, *p* < 0.001, η^2^ = 0.13). Tukey tests indicated bachelor’s > high school for EES (2 > 1); for EAI, postgraduate > bachelor’s and high school (3 > 2; 3 > 1) and bachelor’s > high school (2 > 1). Age showed small negative correlations with EES and EAI (Supplementary Table S3).

### 3.3. Lifestyle-Related Differences in Exercise Enjoyment and Addiction Risk

Sleep status was associated with both EES and EAI (Supplementary Table S4). Post hoc comparisons indicated that EES was higher among participants reporting partly regular sleep than among those reporting regular sleep, whereas other EES contrasts were not significant. For EAI, scores were highest among participants reporting regular sleep, followed by partly regular and irregular sleep, with all pairwise contrasts significant.

Alcohol-use status was associated with EAI but not with EES (Supplementary Table S5). Post hoc comparisons indicated that EAI was higher among occasional alcohol users than among non-users, whereas other contrasts were not significant.

### 3.4. Exercise Participation Differences in Exercise Enjoyment and Addiction Risk

Table 4 indicates exercise frequency strongly differentiated EES (*F* = 43.04, *p* < 0.001, η^2^ = 0.23). Tukey tests indicated 1–2, 3–4, and 5–7 days/week > <1 day/week (2 > 1; 3 > 1; 4 > 1) and 3–4 days/week > 1–2 days/week (3 > 2). EAI did not differ by frequency (*F* = 1.45, *p* > 0.05, η^2^ = 0.01).

Table 5 indicates BMI category strongly differentiated EES (*F* = 53.70, *p* < 0.001, η^2^ = 0.27). Tukey tests indicated normal weight and overweight > underweight and obese (2 > 1; 2 > 4; 3 > 1; 3 > 4). For EAI, the overall difference was borderline (*F* = 2.69, *p* = 0.05) with a very small effect (η^2^ = 0.01); Tukey indicated normal weight > overweight (2 > 3). Exercise type did not differentiate EES but did differentiate EAI (Supplementary Table S6).

### 3.5. Exercise Motives, Training Exposure, and Enjoyment–Addiction Association

Exercise enjoyment was weakly and inversely correlated with exercise addiction-risk scores (*r* = −0.18, *p* = 0.0002; 95% CI: −0.27 to −0.09) (Table 6). Primary exercise motive was associated with both EES and EAI (Supplementary Table S7). Exercise history did not differentiate EES but was associated with EAI (Supplementary Table S8). Single-session exercise duration differentiated both EES and EAI (Supplementary Table S9).

### 3.6. Covariate-Adjusted Association Between Exercise Enjoyment and Addiction Risk

A univariate general linear model predicting exercise enjoyment (EES total score) was significant, *F*(9, 410) = 35.32, *p* < 0.001, explaining 43.7% of the variance in enjoyment (*R*^2^ = 0.437; adjusted *R*^2^ = 0.424). After controlling for age, gender, BMI category, and exercise frequency, exercise addiction (EAI total score) remained a significant negative predictor of enjoyment, *F*(1, 410) = 33.52, *p* < 0.001 (partial η^2^ = 0.076). BMI category and exercise frequency showed large main effects (partial η^2^ = 0.181 and 0.158), whereas age and gender were small but significant predictors (partial η^2^ = 0.013–0.014) (Table 7).

## 4. Discussion

This study investigated whether exercise enjoyment is associated with exercise addiction risk and whether both constructs differ across sociodemographic, lifestyle, and exercise-related characteristics in Turkish adults. The central result was a modest inverse association between enjoyment and addiction risk, indicating that higher enjoyment tended to coincide with slightly lower risk. Because analyses were cross-sectional and most were bivariate (with one covariate-adjusted general linear model (ANCOVA)), this association should be interpreted as correlational and potentially confounded by unmeasured training structure, motives, or lifestyle factors. This pattern is conceptually important because enjoyment and the compulsive features captured by addiction risk are related yet non-identical. In practical terms, enjoyment appears to reflect a generally adaptive experience of exercise, whereas addiction risk reflects a potentially maladaptive persistence pattern that can be maintained even when exercise is no longer consistently pleasurable.

The prevalence of elevated exercise addiction risk in this sample (13.8%; EAI ≥ 24) was within the range reported in prior adult samples, while estimates vary substantially by population and classification approach. Recent multicountry evidence indicates that the proportion classified at risk can differ markedly across settings and criteria, and higher rates are often observed in more exercise-involved groups; therefore, the present estimate should be interpreted as a screening proportion rather than a diagnostic prevalence [15,39,40].

The inverse association can be interpreted as consistent with the notion that compulsive or pressure-driven engagement may carry an affective “cost.” When exercise becomes increasingly rule-bound or internally pressured, enjoyment may diminish despite continued participation [41,42]. The modest association suggests enjoyment is not a consistent protective factor: some people may enjoy exercise yet show risk features (e.g., rigidity), while others may exercise with low enjoyment for instrumental reasons (e.g., health or weight control) [21,43]. Thus, healthy adherence and addiction-like persistence can look similar behaviorally (high involvement) but differ psychologically, especially in flexibility and perceived control versus compulsion and distress when routines are disrupted [20,44].

Interpretation boundaries: Several candidate mechanisms that could explain these patterns were not assessed, including autonomy- versus control-based regulation, harmonious versus obsessive passion, perfectionism, body image concerns, and social or appearance-related pressures. In addition, objective training load, recovery, injury history, and functional impairment were not measured; therefore, elevated screening scores should not be interpreted as evidence of clinical disorder or harm.

Group differences further highlight this distinction. Women reported slightly higher enjoyment but also moderately higher addiction-risk scores than men, suggesting that positive affect and risk can coexist [14,39,45]. This pattern differs from prior Turkish adult evidence in which men showed higher exercise addiction-risk scores than women [3]. This co-occurrence is consistent with a scenario in which exercise is both rewarding and identity-relevant but also more likely to be shaped by social or self-evaluative pressures [14,17,46]. Age showed weak negative associations with both enjoyment and addiction-risk scores, indicating that younger adults may experience stronger affective engagement while also being somewhat more vulnerable to compulsive tendencies [3,47,48]. Education displayed a differentiated pattern: enjoyment was only slightly higher in the mid-education range, whereas addiction risk increased across education levels and peaked among postgraduate participants, implying that achievement-oriented contexts or structured lifestyles may amplify risk even when access to exercise resources is greater [8,49]. Lifestyle-related differences were also observed for sleep patterns and alcohol use; however, interpretations should remain cautious given the cross-sectional design and self-reported measures. Several explanations are plausible (e.g., routine structure, stress exposure, and self-regulation tendencies may differ across groups, or a broader conscientiousness/risk-awareness profile may co-occur with structured training and health monitoring) [16,50]. Regardless of direction, these differences suggest that routine stability and related health behaviors may provide useful contextual markers when interpreting screening scores [51,52].

Sleep status also showed a differentiated profile: enjoyment peaked among partly regular sleepers, whereas addiction-risk scores were highest among regular sleepers. Regular sleep may act as a proxy for more organized daily routines that support training consistency, yet in some individuals the same structure may align with a more rigid, identity-driven exercise pattern [53,54]. Because sleep was assessed coarsely and the sleep–exercise link is likely bidirectional, causal inferences should be avoided [55,56]. Alcohol use was unrelated to enjoyment, but occasional users showed higher addiction-risk scores than non-users, which could reflect lifestyle clustering or compensatory regulation; still, small subgroup sizes and cross-sectional self-report data warrant cautious interpretation [57,58].

Exercise behavior results reinforce that “more exercise” is not synonymous with “addiction risk” [15,21,59]. Enjoyment increased strongly with greater exercise frequency, and those exercising less than once per week reported markedly lower enjoyment, consistent with the idea that positive affect supports sustained participation [60,61]. In contrast, addiction-risk scores did not differ by frequency, suggesting that risk is better indexed by the psychological meaning of exercise and the rigidity of engagement than by participation rate alone [15,21,59]. BMI showed one of the largest gradients for enjoyment, with lower enjoyment among underweight and obese participants, plausibly reflecting differences in comfort, perceived competence, and exercise-related social experience [62,63]. Yet BMI differences in addiction risk were negligible, consistent with addiction risk functioning as a psychological–behavioral tendency rather than a simple reflection of weight status [15,39,59]. Notably, BMI was derived from self-reported height and weight, which may introduce some misclassification [64,65].

Exercise type did not differentiate enjoyment but did differentiate addiction risk, with higher scores in flexibility-based exercise compared with strength and cardio categories [15,39]. This pattern should not be taken to imply that any modality is inherently risky; rather, it suggests that the profiles of individuals selecting specific activities, along with their motives, norms, and self-regulatory styles, may shape risk [14,39]. Motives offered the clearest psychological separation: performance-enhancement motives aligned with higher enjoyment, whereas addiction risk was higher among those endorsing motives such as weight loss/control and stress management [39,66]. This motive pattern supports the view that controlled or compensatory motives may foster rigidity and persistence under internal pressure, whereas more mastery-oriented motives may be more compatible with enjoyment-driven engagement [43,67].

Finally, addiction risk was highest in the mid-duration exercise-history category and increased with longer single-session durations, peaking in the longest-duration group [16,68]. Together, these findings suggest that duration and entrenched routines may be more sensitive behavioral markers of risk than weekly frequency [40,69]. This has applied relevance: practitioners may wish to attend not only to how often individuals exercise but also to the structure, flexibility, and underlying drivers of their routines.

## 5. Limitations

The cross-sectional design prevents causal inference and does not clarify temporal ordering. All measures were self-reported; BMI was derived from self-reported height and weight (which can underestimate BMI and misclassify BMI categories), and addiction was indexed using a screening tool that identifies elevated risk rather than clinical diagnosis. Several contextual variables (e.g., sleep status, alcohol use, and exercise motives) were assessed with brief, categorical self-report items; therefore, measurement precision is limited, misclassification is possible, and associations (including covariate-adjusted effects) may be attenuated or affected by residual confounding. The sample was recruited via online convenience methods and was predominantly young adults (mean age ≈ 25.7 years), which may limit generalizability to older or less active populations. In addition, multiple subgroup comparisons were conducted without a formal multiplicity correction; accordingly, some statistically significant findings may reflect chance, and interpretation should prioritize effect sizes and consistency of patterns. Additional multivariable models (including models with addiction risk as the outcome) are warranted to evaluate independent associations after accounting for training characteristics and motives. Future research would benefit from longitudinal designs, richer assessment of psychological mechanisms (e.g., self-regulation, perfectionism, body-related concerns, need satisfaction), and more objective indicators of training load and impairment (e.g., injury history, functional interference) to distinguish high commitment from harmful compulsion more precisely. Given the bounded nature of the EAI score and small subgroup sizes in some categories (e.g., obesity), robust and/or non-parametric sensitivity analyses may be informative. Most subgroup analyses remained unadjusted for covariates; however, one covariate-adjusted general linear model (ANCOVA) was conducted to predict exercise enjoyment.

## 6. Conclusions

In Turkish adults, exercise enjoyment was modestly associated with lower exercise addiction risk, indicating partial divergence between pleasurable, adaptive engagement and risk-linked, potentially compulsive persistence. Enjoyment was most strongly patterned by exercise frequency and BMI, whereas addiction risk varied more consistently with factors reflecting structure, motives, and training load (e.g., sleep regularity, exercise motives, session duration, and exercise history). Because the study was cross-sectional and subgroup analyses were exploratory, these findings should be interpreted as descriptive associations. Future work using longitudinal designs and covariate-adjusted models is needed to clarify temporal ordering and to identify which motivational and behavioral features best differentiate high commitment from clinically meaningful impairment.

## Figures and Tables

**Table 1:** Distribution of participants by selected demographic variables (*N* = 420).

Variable	Category	*n*	%
Gender	Female	189	45.0
	Male	231	55.0
Marital status	Married	183	43.6
	Single	237	56.4
Employment status	Employed	355	84.5
	Unemployed	65	15.5
Education level (highest completed)	High school	179	42.6
	Bachelor’s degree	211	50.2
	Postgraduate degree	30	7.1

Note. Participants had a mean age of 25.68 years (*SD* = 5.60).

**Table 2:** Independent-samples *t*-test results for EES and EAI by gender (*N* = 420).

Variable	Female (*n* = 189) *M*	Female *SD*	Male (*n* = 231) *M*	Male *SD*	*t*	*df*	*p*	Cohen’s *d*
EES	49.32	6.67	47.34	9.27	2.45	418	0.01 *	0.24
EAI	19.08	4.93	16.20	5.43	5.63	418	<0.001 *	0.55

Note. EES = Exercise Enjoyment Scale; EAI = Exercise Addiction Inventory; *M* = mean; *SD* = standard deviation; *df* = degrees of freedom; *d* = Cohen’s d. * *p* < 0.05.

**Table 3:** One-way ANOVA results for EES and EAI by education level (*N* = 420).

Variable	Group	*n*	*M*	*SD*	*F*	*p*	Tukey	η^2^
EES	1. High school	179	47.05	9.50	4.23	0.01 *	2 > 1	0.02
	2. Bachelor’s	211	49.39	6.95				
	3. Postgraduate	30	47.16	7.83				
	Total	420	48.23	8.26				
EAI	1. High school	179	15.56	4.87	31.69	<0.001 *	3 > 2; 3 > 1; 2 > 1	0.13
	2. Bachelor’s	211	18.43	5.42				
	3. Postgraduate	30	22.53	2.80				
	Total	420	17.50	5.40				

Note. EES = Exercise Enjoyment Scale; EAI = Exercise Addiction Inventory; *M* = mean; *SD* = standard deviation; η^2^ = eta squared. Tukey codes indicate significant pairwise differences (group numbers correspond to the order listed). * *p* < 0.05.

**Table 4:** One-way ANOVA results for EES and EAI by exercise frequency (*N* = 420).

Variable	Group	*n*	*M*	*SD*	*F*	*p*	Tukey	η^2^
EES	1. Less than once/week	25	32.84	7.24	43.04	<0.001 *	4 > 1; 3 > 1; 3 > 2; 2 > 1	0.23
	2. 1–2 days/week	93	47.30	7.39				
	3. 3–4 days/week	244	49.94	6.98				
	4. 5–7 days/week	58	49.17	8.03				
	Total	420	48.23	8.26				
EAI	1. Less than once/week	25	16.92	5.21	1.45	0.22	None	0.01
	2. 1–2 days/week	93	18.51	5.29				
	3. 3–4 days/week	244	17.28	5.48				
	4. 5–7 days/week	58	17.06	5.27				
	Total	420	17.50	5.40				

Note. EES = Exercise Enjoyment Scale; EAI = Exercise Addiction Inventory; *M* = mean; *SD* = standard deviation; η^2^ = eta squared. Tukey codes indicate significant pairwise differences (group numbers correspond to the order listed). * *p* < 0.05.

**Table 5:** One-way ANOVA results for EES and EAI by BMI category (*N* = 420).

Variable	Group	*n*	*M*	*SD*	*F*	*p*	Tukey	η^2^
EES	1. Underweight	22	34.31	9.67	53.70	<0.001 *	2 > 1; 2 > 4; 3 > 1; 3 > 4	0.27
	2. Normal weight	267	49.37	6.99				
	3. Overweight	111	50.41	6.65				
	4. Obese	20	36.30	6.34				
	Total	420	48.23	8.26				
EAI	1. Underweight	22	17.31	5.47	2.69	0.05 *	2 > 3	0.01
	2. Normal weight	267	18.01	5.36				
	3. Overweight	111	16.32	5.48				
	4. Obese	20	17.45	4.78				
	Total	420	17.50	5.40				

Note. EES = Exercise Enjoyment Scale; EAI = Exercise Addiction Inventory; *M* = mean; *SD* = standard deviation; η^2^ = eta squared. Tukey codes indicate significant pairwise differences (group numbers correspond to the order listed). * *p* < 0.05.

**Table 6:** Correlation between EES and EAI (*N* = 420).

Variables	*n*	EES	EAI
EES	420	—	−0.18 **
EAI	420	−0.18 **	—

Note. EES = Exercise Enjoyment Scale; EAI = Exercise Addiction Inventory; values are Pearson’s *r*. ** *p* < 0.01 (two-tailed).

**Table 7:** Results of the univariate general linear model predicting exercise enjoyment (EES total score) (*N* = 420).

Variable	Type III SS	*df*	*MS*	*F*	*p*	Partial η^2^
Gender	231.884	1	231.884	5.903	0.016	0.014
BMI (categorical)	3558.847	3	1186.282	30.200	<0.001	0.181
Exercise frequency (categorical)	3013.792	3	1004.597	25.575	<0.001	0.158
Age	205.426	1	205.426	5.230	0.023	0.013
Exercise addiction (total)	1316.629	1	1316.629	33.519	<0.001	0.076
Error	16,105.045	410	39.281			

Note. Type III sums of squares are reported. Partial η^2^ = partial eta squared. BMI category and exercise frequency were entered as categorical factors; age and exercise addiction (EAI total score) were entered as covariates.

## Data Availability

The data presented in this study are available upon request from the corresponding author due to confidentiality concerns.

## Supplementary Materials

The following supporting information can be downloaded at: https://www.mdpi.com/article/10.3390/healthcare14060703/s1, Table S1. Independent-samples *t*-test results for EES and EAI by marital status. Table S2. Independent-samples *t*-test results for EES and EAI by employment status. Table S3. Correlations of age with EES and EAI. Table S4. One-way ANOVA results for EES and EAI by sleep status. Table S5. One-way ANOVA results for EES and EAI by alcohol use status. Table S6. One-way ANOVA results for EES and EAI by exercise type. Table S7. One-way ANOVA results for EES and EAI by primary exercise motive. Table S8. One-way ANOVA results for EES and EAI by exercise history (duration of exercising). Table S9. One-way ANOVA results for EES and EAI by single-session exercise duration.

## Abbreviations

The following abbreviations are used in this manuscript:

BMI	Body Mass Index
EAI	Exercise Addiction Inventory
EES	Exercise Enjoyment Scale
