# Supplementary material for: Exercise Enjoyment and Exercise Addiction Risk Among Turkish Adults: Associations and Subgroup Differences in a Cross-Sectional Survey
Source: Healthcare (Basel). 2026 Mar 10;14(6):703. doi: 10.3390/healthcare14060703 (PMC13026342; doi:10.3390/healthcare14060703)
Supplement: Supplementary file 1 [file healthcare-14-00703-s001.zip › healthcare-4166355-supplementary.pdf]

Supplementary Materials

Supplementary Table S1. Independent-samples t-test results for EES and EAI by marital status (N = 420).

Variable	Married (n = 183) M	Married SD	Single (n = 237) M	Single SD	t	df	p	Cohen's d
EES	47.66	8.24	48.67	8.25	−1.25	418	.21	−0.12
EAI	17.59	5.14	17.43	5.60	0.30	418	.76	0.03

Note. EES = Exercise Enjoyment Scale; EAI = Exercise Addiction Inventory; M = mean; SD = standard deviation; df = degrees of freedom; d = Cohen's d. * $p < .05$.

Supplementary Table S1. indicates no marital-status differences in EES (t(418) = −1.25, p > .05, d = −0.12; married: M = 47.66, SD = 8.24; single: M = 48.67, SD = 8.25) or EAI (t(418) = 0.30, p > .05, d = 0.03; married: M = 17.59, SD = 5.14; single: M = 17.43, SD = 5.60).

Supplementary Table S2. Independent-samples t-test results for EES and EAI by employment status (N = 420).

Variable	Employed (n = 355) M	Employed SD	Unemployed (n = 65) M	Unemployed SD	t	df	p	Cohen's d
EES	48.37	8.19	47.47	8.64	0.80	418	.42	0.10
EAI	17.63	5.36	16.80	5.63	1.14	418	.25	0.15

Note. EES = Exercise Enjoyment Scale; EAI = Exercise Addiction Inventory; M = mean; SD = standard deviation; df = degrees of freedom; d = Cohen's d. * $p < .05$.

Supplementary Table S2 indicates no employment-status differences in EES (t(418) = 0.80, p > .05, d = 0.10; employed: M = 48.37, SD = 8.19; unemployed: M = 47.47, SD = 8.64) or EAI (t(418) = 1.14, p > .05, d = 0.15; employed: M = 17.63, SD = 5.36; unemployed: M = 16.80, SD = 5.63).

Supplementary Table S3. Correlations of age with EES and EAI (N = 420).

Variable	n	EES	EAI
Age	420	−0.14**	−0.22**

Note. EES = Exercise Enjoyment Scale; EAI = Exercise Addiction Inventory; values are Pearson's r. ** $p < .01$ (two-tailed).

Supplementary Table S3 indicates age showed small negative correlations with EES (r = −0.14, p < .01) and EAI (r = −0.22, p < .01), indicating slightly lower enjoyment and lower addiction-risk scores at higher ages.

Supplementary Table S4. One-way ANOVA results for EES and EAI by sleep status (N = 420).

Variable	Group	n	M	SD	F	p	Tukey	η^2
EES	1. Regular sleep	190	47.13	8.16	4.83	.008*	2 > 1	0.02
	2. Partly regular	162	49.79	7.07				
	3. Irregular	68	47.60	10.41				
	Total	420	48.23	8.26				
EAI	1. Regular sleep	190	19.35	4.64	31.52	< .001*	1 > 2; 1 > 3; 2 > 3	0.13
	2. Partly regular	162	16.84	5.33				
	3. Irregular	68	13.89	5.41				
	Total	420	17.50	5.40				

Note. EES = Exercise Enjoyment Scale; EAI = Exercise Addiction Inventory; M = mean; SD = standard deviation; η^2 = eta squared. Tukey codes indicate significant pairwise differences (group numbers correspond to the order listed). * $p < .05$.

Supplementary Table S4 indicates sleep status was associated with EES ($F = 4.83$, $p = .008$, $\eta^2 = 0.02$) and EAI ($F = 31.52$, $p < .001$, $\eta^2 = 0.13$). Tukey tests indicated partly regular > regular for EES (2 > 1); for EAI, regular > partly regular and irregular (1 > 2; 1 > 3) and partly regular > irregular (2 > 3).

Supplementary Table S5. One-way ANOVA results for EES and EAI by alcohol use status (N = 420).

Variable	Group	n	M	SD	F	p	Tukey	η^2
EES	1. Uses alcohol	38	46.92	8.84	1.97	.14	None	0.00
	2. Does not use	262	48.85	8.16				
	3. Occasionally	120	47.30	8.20				
	Total	420	48.23	8.26				
EAI	1. Uses alcohol	38	18.02	5.62	16.31	< .001*	3 > 2	0.07
	2. Does not use	262	16.42	5.43				
	3. Occasionally	120	19.69	4.57				
	Total	420	17.50	5.40				

Note. EES = Exercise Enjoyment Scale; EAI = Exercise Addiction Inventory; M = mean; SD = standard deviation; η^2 = eta squared. Tukey codes indicate significant pairwise differences (group numbers correspond to the order listed). * $p < .05$.

Supplementary Table S5 indicates alcohol-use status was not associated with EES ($F = 1.97$, $p > .05$) but was associated with EAI ($F = 16.31$, $p < .001$, $\eta^2 = 0.07$), with occasional users scoring higher than non-users (3 > 2).

Supplementary Table S6. One-way ANOVA results for EES and EAI by exercise type (N = 420).

Variable	Group	n	M	SD	F	p	Tukey	η^2
EES	1. Cardio (running, swimming, cycling, etc.)	149	49.30	7.44	1.97	.14	None	0.00
	2. Strength training (weights, etc.)	158	47.56	9.79				
	3. Flexibility (yoga, pilates, etc.)	113	47.76	6.71				
	Total	420	48.23	8.26				
EAI	1. Cardio (running, swimming, cycling, etc.)	149	15.45	5.58	32.90	< .001*	3 > 2; 3 > 1; 2 > 1	0.13
	2. Strength training (weights, etc.)	158	17.27	5.07				
	3. Flexibility (yoga, pilates, etc.)	113	20.53	4.13				
	Total	420	17.50	5.40				

Note. EES = Exercise Enjoyment Scale; EAI = Exercise Addiction Inventory; M = mean; SD = standard deviation; η^2 = eta squared. Tukey codes indicate significant pairwise differences (group numbers correspond to the order listed). * $p < .05$.

Supplementary Table S6 indicates EES did not differ by exercise type ($F = 1.97$, $p > .05$), whereas EAI differed across types ($F = 32.90$, $p < .001$, $\eta^2 = 0.13$). Tukey tests indicated flexibility-based > strength training and cardio (3 > 2; 3 > 1) and strength training > cardio (2 > 1).

Supplementary Table S7. One-way ANOVA results for EES and EAI by primary exercise motive (N = 420).

Variable	Group	n	M	SD	F	p	Tukey	η^2
EES	1. Weight loss/control	90	47.28	7.77	3.39	.01*	2 > 1; 2 > 3; 2 > 4	0.03
	2. Performance enhancement	146	50.11	7.53				
	3. Health & fitness	113	47.31	9.31				
	4. Stress management	36	45.80	9.46				
	5. Socialization	35	48.28	6.03				
	Total	420	48.23	8.26				
EAI	1. Weight loss/control	90	19.68	4.89	13.32	< .001*	1 > 2; 1 > 3; 4 > 2; 4 > 3; 5 > 2; 5 > 3	0.11
	2. Performance enhancement	146	16.28	5.28				
	3. Health & fitness	113	15.83	5.56				

	4. Stress management	36	20.47	3.51
	5. Socialization	35	19.31	4.73
	Total	420	17.50	5.40

Note. EES = Exercise Enjoyment Scale; EAI = Exercise Addiction Inventory; M = mean; SD = standard deviation; η^2 = eta squared. Tukey codes indicate significant pairwise differences (group numbers correspond to the order listed). * $p < .05$.

Supplementary Table S7 indicates exercise motive was associated with EES ($F = 3.39$, $p < .05$, $\eta^2 = 0.03$) and EAI ($F = 13.32$, $p < .001$, $\eta^2 = 0.11$). Tukey tests indicated performance enhancement > weight loss/control, health & fitness, and stress management for EES (2 > 1; 2 > 3; 2 > 4); for EAI, weight loss/control, stress management, and socialization > performance enhancement and health & fitness (1 > 2; 1 > 3; 4 > 2; 4 > 3; 5 > 2; 5 > 3).

Supplementary Table S8. One-way ANOVA results for EES and EAI by exercise history (duration of exercising) (N = 420).

Variable	Group	n	M	SD	F	p	Tukey	η²
EES	1. Up to 0–1 year	74	47.37	9.38	0.50	.68	None	0.00
	2. 1–3 years	184	48.22	8.90				
	3. 3–5 years	124	48.42	6.56				
	4. More than 5 years	38	49.31	7.78				
	Total	420	48.23	8.26				
EAI	1. Up to 0–1 year	74	15.39	5.16	14.91	< .001*	3 > 1; 3 > 2; 3 > 4	0.09
	2. 1–3 years	184	16.82	6.00				
	3. 3–5 years	124	19.97	3.70				
	4. More than 5 years	38	16.86	4.72				
	Total	420	17.50	5.40				

Note. EES = Exercise Enjoyment Scale; EAI = Exercise Addiction Inventory; M = mean; SD = standard deviation; η^2 = eta squared. Tukey codes indicate significant pairwise differences (group numbers correspond to the order listed). * $p < .05$.

Supplementary Table S8 indicates exercise history did not differentiate EES ($F = 0.50$, $p > .05$, $\eta^2 = 0.00$), whereas EAI differed across history groups ($F = 14.91$, $p < .001$, $\eta^2 = 0.09$). Tukey tests indicated the 3–5 year group > 0–1 year, 1–3 years, and >5 years (3 > 1; 3 > 2; 3 > 4).

Supplementary Table S9. One-way ANOVA results for EES and EAI by single-session exercise duration (N = 420).

Variable	Group	n	M	SD	F	p	Tukey	η²
EES	1. 0–30 min	31	41.00	9.80	10.72	< .001*	2 > 1; 3 > 1; 4 > 1	0.07

	2. 31–60 min	181	48.31	8.69				
	3. 61–90 min	184	49.61	6.70				
	4. > 91 min	24	46.41	8.92				
	Total	420	48.23	8.26				
EAI	1. 0–30 min	31	15.93	4.28	7.17	< .001*	3 > 1; 3 > 2; 4 > 1; 4 > 2; 4 > 3	0.04
	2. 31–60 min	181	16.66	5.32				
	3. 61–90 min	184	18.11	5.45				
	4. > 91 min	24	21.20	4.87				
	Total	420	17.50	5.40				

Note. EES = Exercise Enjoyment Scale; EAI = Exercise Addiction Inventory; M = mean; SD = standard deviation; η^2 = eta squared. Tukey codes indicate significant pairwise differences (group numbers correspond to the order listed). * $p < .05$.

Supplementary Table S9 indicates Single-session duration differentiated EES ($F = 10.72$, $p < .001$, $\eta^2 = 0.07$) and EAI ($F = 7.17$, $p < .001$, $\eta^2 = 0.04$). Tukey tests indicated 0–30 min < 31–60, 61–90, and >91 min for EES (2 > 1; 3 > 1; 4 > 1). For EAI, 61–90 min and >91 min > 0–30 min and 31–60 min, and >91 min > 61–90 min (3 > 1; 3 > 2; 4 > 1; 4 > 2; 4 > 3).
